# Viral Infection and Antiviral Treatments in Ocular Pathologies

**DOI:** 10.3390/microorganisms10112224

**Published:** 2022-11-10

**Authors:** Francesco Petrillo, Arianna Petrillo, Francesca Paola Sasso, Antonietta Schettino, Angela Maione, Marilena Galdiero

**Affiliations:** 1 Azienda Ospedaliera Universitaria-Città della Salute e della Scienza di Torino, 10126 Torino, Italy; 2 Università Degli Studi di Milano, 20122 Milan, Italy; 3 Università Degli Studi La Sapienza, 00185 Rome, Italy; 4 Department of Experimental Medicine, University of Campania “Luigi Vanvitelli”, 80138 Naples, Italy; 5 Department of Biology, University of Naples Federico II, 80126 Naples, Italy

**Keywords:** antiviral drug, corticosteroids, viral infection, viruses

## Abstract

Ocular viral infections are common and widespread globally. These infectious diseases are a major cause of acute red eyes and vision loss. The eye and its nearby tissues can be infected by several viral agents, causing infections with a short course and limited ocular implications or a long clinical progression and serious consequences for the function and structure of the ocular region. Several surveillance studies underline the increased emergence of drug resistance among pathogenic viral strains, limiting treatment options for these infections. Currently, in the event of resistant infections, topical or systemic corticosteroids are useful in the management of associated immune reactions in the eye, which contribute to ocular dysfunction. Many cases of viral eye infections are misdiagnosed as being of bacterial origin. In these cases, therapy begins late and is not targeted at the actual cause of the infection, often leading to severe ocular compromises, such as corneal infiltrates, conjunctival scarring, and reduced visual acuity. The present study aims at a better understanding of the viral pathogens that cause eye infections, along with the treatment options available.

## 1. Introduction

The eye is a spheroidal organ located in the orbital cavity and protected by the eyelids and other ocular annexes [1]. Structurally, the eye consists of an internal compartment formed by the anterior and posterior chambers, iris, lens, vitreous cavity, retina, ciliary body, choroid, and intrinsic ocular muscles; and of an external compartment comprising the conjunctiva, the cornea, the sclera, and the tear film [2]. The internal compartment, physically separated from the immune system by the eye’s blood–retinal barrier, maintains a sterile environment [3]. In contrast, the outer compartment of the eye is exposed to the external environment and, therefore, is susceptible to contamination with potentially pathogenic microorganisms [4,5,6]. Mechanical, anatomical, and immunological defense mechanisms have evolved to protect the ocular surface from many external agents [7]. The stability potential of the eye plays a central role in repelling aerosol particles near the surface of the eye [8]. In order to prevent attack by pathogenic microbial species, the eye possesses several defense mechanism, such as the production of a tear film containing several antimicrobial components—including lactoferrin, defensins, and lysozymes—that help to prevent colonization by pathogens, the presence of the conjunctiva as a physical and biological barrier to external environments, the existence of an innate immune response of the ocular surface epithelium that recognizes only potential pathogens’ antigens and, finally, the presence of a commensal microbiota that constitutes the “barrier population” against pathogenic microbes [9,10,11]. The colonization of the ocular surface by commensal microorganisms has been confirmed by several studies [12,13]. The ocular surface of healthy individuals possesses a native microbiome that includes both viral and bacterial communities [14]. The ocular microbiota is widely known. Several studies have revealed a considerable complexity in the composition of the ocular microbiota, consisting of about 221 bacterial species. The most abundant phyla are represented by Proteobacteria, Actinobacteria, and Firmicutes [12,15]. Few studies have reported on the ocular virome. In the vitreous fluids of healthy individuals, the major viral families found include Myoviridae, Siphoviridae, Phycodnaviridae, Herpesviridae, Poxviridae, Iridoviridae, Podoviridae, Retroviridae, Baculoviridae, and Flaviviridae [14]. Viral and bacterial agents coexist in equilibrium with the host in immunocompetent individuals and play a crucial role in maintaining the homeostasis of the eye’s surface. The alteration of this ecosystem through various routes—such as poor hand hygiene, traumatic injuries, surgery, the transplacental route, the improper use of contact lenses, or the anatomical proximity and cellular receptor distribution between ocular and respiratory or nervous tissues—can promote the invasion of potentially pathogenic external microorganisms [16,17] and the alteration of the microbiota.

The majority of eye infections are attributed to bacteria, although viruses, fungi, and parasites can also contribute to ocular pathogenesis [18,19]. Among Gram-negative bacteria, the genus *Pseudomonas* is the most represented, while among the Gram-positive strains, Staphylococci are of considerable importance [20,21]. *Fusarium* spp. infections and *Candida albicans* and *Aspergillus* spp. endophthalmitis are also worth mentioning. With regard to viral infections, respiratory viruses such as human adenovirus (species D), avian influenza virus (H7), herpesviruses, coronaviruses, and arboviruses can cause conjunctivitis and/or keratoconjunctivitis [22,23]. The conjunctiva is therefore considered an optimal route of entry for respiratory viruses, and infected tears and conjunctival secretions can lead to the spread of infections [24]. The purpose of this review is to describe the different pathogens that can cause eye infections, focusing on viral infections and the role of viruses in the pathogenesis of eye infections. Therefore, knowing how to discriminate between the various microorganisms that can cause eye infections can enable targeted interventions to treat or prevent this type of infection.

## 2. Ocular Infection

### Types of Ocular Infection

Blepharitis is an inflammation that affects the eyelid margin at the level of the eyelash implant; rather frequently, it tends to become chronic and relapse. Different forms have been distinguished, including hyperemic, seborrheic, scaly, and ulcerative blepharitis. The etiology is often bacterial. It is an infection of the ciliary follicle, often caused by *Staphylococcus aureus* or *S. epidermidis*, but sometimes it can be secondary to the presence of a mite (*Demodex folliculorum*), followed by an allergic reaction and secondary infection by bacteria that invade the hair follicle [25].

Dacryocystitis is an infection of the lacrimal sac, usually secondary to an inflammatory process of the nasal meatus or to a patency defect of the nasolacrimal duct that leads to stagnation of tear fluid. The onset of symptoms consists of copious tearing and conjunctival hyperemia, with discharge of purulent material from the lacrimal points; there may also be swelling of the lacrimal sac area [26].

Conjunctivitis is an inflammatory process of the mucous membrane that covers a large part of the anterior aspect of the eyeball and the posterior surface of the eyelids. The conjunctival sac, directly exposed to the external environment, is easily attacked by irritating factors (e.g., chemical, physical, allergens), but above all by infectious agents. Infectious conjunctivitis is very common and represents one of the most important eye diseases. The occurrence of this infectious state can be a consequence of certain erroneous behaviors (e.g., close contact with infected individuals, the possibility of touching contaminated hands, etc.) that expose the ocular conjunctiva to pathogens. A variety of evidence indicates that the rate of bacterial conjunctivitis is about 50%. Another study reported that bacteria are responsible for only 50% of cases of suspected bacterial conjunctivitis. Conversely, a study reported that up to 52% of cases treated as viral conjunctivitis turned out to be bacterial conjunctivitis following the culture examination [27].

Keratitis is an inflammatory process of the corneal tissue. Excluding forms caused by physical (e.g., UV rays) and chemical agents (e.g., acids and alkalis), most cases of keratitis (about 80%) have an infectious etiology. The causative agents of infection can be bacteria, viruses, fungi, and/or protozoa. Currently, in countries where water purified of biological pollutants is available, infections with pyogenic bacteria are rare. However, because of the spread of soft contact lenses, the frequency of infectious keratitis caused by bacteria and fungi that contaminate the containers and liquids used for storage has increased [28]. 

Trachoma is a severe form of chronic keratoconjunctivitis and is the leading cause of blindness in some developing countries. It is caused by *Chlamydia trachomatis*, and its defining pathological characteristic is the formation of inclusion bodies called Halberstaedter–Prowazek bodies [29].

Infectious scleritis is an isolated acute inflammation of the sclera—the opaque fibrous membrane that makes up five-sixths of the outer tunic of the eyeball. Clinically, it manifests with severe pain and red eye. Scleritis and episcleritis are generally rarely caused by infectious agents. Despite this, diffuse scleritis is reported in some cases of tuberculosis and herpes zoster. Episcleritis generally has a benign, self-limited course, while scleritis can result in a thinning of the sclera and, sometimes, in staphyloma [30].

Infectious endophthalmitis is a suppurative process of the vitreous body, with a tendency to abscess and spread to other eye districts. The responsible microorganisms are mainly bacteria, but also fungi [31]. Among the Gram-positive bacteria, coagulase-negative Staphylococci, *S. aureus*, *Streptococcus* spp., and *Enterococcus* spp. are the leading causes of endophthalmitis. Of the Gram-negative bacteria, *Pseudomonas* spp., *K. pneumoniae*, and *E. coli* are the most representative. Of the fungi involved in these pathologies, the genera *Candida*, *Fusarium*, and *Aspergillus* are involved in endophthalmitis cases. 

Uveitis is an inflammation state of part or all of the middle (vascular) layer of the ocular wall. The inflammatory phenomena can be of an autoimmune or infectious nature, with simultaneous involvement of the adjacent ocular portions (i.e., the sclera, cornea, and retina). In particular, it is possible to distinguish (i) anterior uveitis, which can affect the iris (iritis), the ciliary body (cyclitis), or both (iridocyclitis); and (ii) posterior uveitis, which can involve the choroid (choroiditis), the retina (retinitis), or both (chorioretinitis). Sometimes, retinal vasculitis with possible involvement is also observed in the posterior vitreous body [32]. The sites of ocular infections and associated etiological agents were shown in Table 1.

## 3. Ocular Infections Caused by Viruses

Viral infections of the ocular surface are a major public health problem worldwide. Research published in the last decade indicates that in the United States and the United Kingdom most eye exams were related to conjunctivitis (allergic, infective, or chemical) [8]. Among these, the most frequent type is infective conjunctivitis, and it is known that the most common etiological agents are viruses (up to 80% of all cases) [33]. Many cases are misdiagnosed as bacterial conjunctivitis [34]. Some studies report failure to diagnose the causative agent of viral conjunctivitis in fewer than 50% of cases [8,27]. In these cases, obviously, the therapy is started late, and the proper causative agent is not targeted, so some patients develop corneal infiltrates, conjunctival scars, and reduced visual acuity. It has emerged that up to 90% of cases of viral conjunctivitis are caused by adenoviruses [35]. 

In addition to adenoviruses, the herpes simplex virus (which is the cause of 1.3–4.8% of all cases of acute conjunctivitis), enterovirus D70, and coxsackievirus A24 have also been isolated from the conjunctiva and implicated in the development of conjunctivitis [8]. Varicella (herpes)-zoster virus (VZV) and *Molluscum contagiosum* can also be responsible for viral conjunctivitis. Additionally, there are ocular infections caused by respiratory viruses, which are also frequent [36]. In fact, respiratory viruses such as adenoviruses, influenza viruses, respiratory syncytial viruses, coronaviruses, and rhinoviruses are the most common causative agents of several diseases in humans, with a spectrum ranging from mild influenza symptoms to severe respiratory failure [37]. The main transmission route of respiratory viruses is by inhalation of infected aerosols or by contact with contaminated environmental surfaces [38]. 

### 3.1. Types of Ocular Infection

#### 3.1.1. Respiratory Viruses

The ocular surface can be exposed to infectious aerosols; therefore, respiratory viruses, although generally considered respiratory pathogens, are also capable of causing eye infections in infected individuals or inducing a respiratory infection after ocular exposure [39]. Several respiratory viruses show a particular ocular tropism, which gives the eye the property of acting as a perfect viral replication site but also as a portal of entry for viruses in extraocular sites to induce respiratory infections [40]. The main anatomical link between ocular and respiratory tissues is represented by the nasolacrimal system. Indeed, the tear duct transports the tear fluid from the ocular surface to the lower meatus of the nose, facilitating the transfer of viruses to the tissue of the respiratory tract, but at the same time the epithelium of the nasolacrimal duct, through the microvilli, can allow the secretion and reabsorption of tear fluids’ components [41]. For this reason, the nasolacrimal duct can serve as a link for the exchange of potentially virus-infected fluids. In addition to the anatomical proximity between the ocular and respiratory tissues, the cellular receptor distribution between the ocular and respiratory systems can also contribute to the tropism of respiratory viruses [42]. For example, the human respiratory and ocular tissues’ epithelial cells contain terminal sialic acid (SA) glycoproteins. These recognize and bind to viral glycoproteins, promoting viral entry and the consequent initiation of the infectious process [43]. The nasal mucosa and trachea contain the α2-6 ASs, while in the lower respiratory tract and in the ocular tissues α2-3 ASs are found in greater abundance [44]. It is interesting to underline that the epithelia of the lacrimal sac and the nasolacrimal duct, which form connections between ocular and respiratory tissues, express both types of SA, thereby facilitating the passage of viruses with a particular tropism for the ocular tissues to the respiratory tissues, and vice versa. The distribution of receptors in ocular and respiratory tissues is closely related to the tropism of numerous respiratory viruses [41]. 

Adenoviruses, isolated from human adenoids in 1953 by Rowe et al., are non-enveloped, double-stranded DNA (composed of 30,000 to 38,000 base pairs) viruses with icosahedral capsids [44]. Adenoviruses can cause upper respiratory tract infections, pneumonia, adenitis, myocarditis, encephalitis, gastroenteritis, and meningitis. Moreover, they are responsible for 65% to 90% of all cases of viral conjunctivitis [45]. The virus uses surface receptors—including CD46, sialic acid, coagulation factor IX, dipalmitoyl phosphatidylcholine, and lactoferrin—present on the host cell for initial binding [46]. After binding to the surface receptors, the virus is absorbed into the cell via endocytosis. The virus begins its replication using the host’s transcription mechanisms [47]. After producing the viral proteins, the assembly of viral particles takes place within the nucleus and cytoplasm. Adenoviruses in epithelial cells are able to complete the replication cycle, induce cell death, and release the viral progeny. In lymphoid cells, on the other hand, they cause a latent infection, and only a small amount of the viral progeny is released [48]. The virus can be transmitted through tears, feces, saliva, and respiratory droplets from infected persons, but also by using infected personal items or by direct contact with contaminated fingers. Infected individuals can shed viral particles for up to 14 days after the onset of symptoms [49]. Consequently, frequent hand washing, the use of gloves, and cleaning of personal items are crucial in order to reduce transmission. Ocular manifestations of adenoviruses include epidemic keratoconjunctivitis (EKC), pharyngoconjunctival fever (PCF), and nonspecific conjunctivitis [50]. One study reported rare cases of adenoviral conjunctivitis that generated orbital inflammation with restriction of extraocular motility and diplopia [51]. Although adenoviral conjunctivitis is generally considered to be a disease without serious consequences, significant, life-changing complications can occur in some cases, including bulbar and conjunctival scarring, symblepharon formation, nummular keratitis, and dry eye secondary to fibrosis in the lacrimal gland and ducts [35].

Human influenza viruses belong to the Orthomixoviridae family. They are enveloped, their nucleocapsid has helical symmetry, and their genome is made up of single-stranded RNA organized in segments. Influenza viruses that infect humans are organized into three strains: A, B, and C. Respiratory infections typically cause infections that can also involve other organs, such as the eyes [52]. The virus preferentially binds to α2-6 ASs receptors; therefore, it is able to replicate mainly in the upper respiratory tract, while avian influenza viruses bind to α2-3 ASs receptors and replicate in the lower respiratory tract tissue, where these receptors are more abundant [53]. As for adenoviruses, those with respiratory tropism bind to the following receptors: CD46, desmoglein-2 (DSG-2), or the coxsackievirus and adenovirus receptor (CAR); while adenovirus serotypes with a particular ocular tropism use α2-3-linked SA receptors and GD1a located on the ocular surface [41]. Several studies have shown that a wide range of human and avian influenza viruses are also capable of replicating in different types of ocular cells in vitro and can effectively use ocular exposure to trigger a productive respiratory infection in vivo [54]. It is reasonable to assume similar occupational exposure during culling and depopulation activities necessitated by H5 or H7 infection in poultry; however, reports of ocular complications predominate only among workers exposed to H7 viruses, indicating that not all subtypes of viruses seem to exploit this route of entry into humans in the same way [55]. Only a fraction of all human influenza virus infections involve ocular complications. Although understanding of the binding profile of the H7 hemagglutinin receptor has improved in recent years, ocular tropism is unlikely to be governed solely by this property [53]. Indeed, it is safe to assume that the exposure of the respiratory tract tissue—and not the ocular tissue—to the influenza virus may represent a mode of transmission of the infection. The ocular mucosal surface protection prevents the influenza virus from potentially exploiting a mucosal surface with an anatomical conduit to the nasopharynx and respiratory tract [56]. It is therefore always recommended to use eye protection in those circumstances where there is a risk of influenza virus infection. However, further studies are needed to understand how these pathogens are able to enter the respiratory tract and cause ocular complications. Furthermore, the distribution of the angiotensin-converting enzyme 2 (ACE2) receptor in different tissues—such as ocular, heart, and lung tissues—is related to severe respiratory disease and ocular manifestations associated with coronavirus infections [57]. Despite advances in our understanding of the ocular tropism of respiratory pathogens, there is still work to be done for a more complete understanding of the properties conferring this tropism and how to best prevent human eye diseases caused by these etiological agents [58]. 

Rhinoviruses belong to the Picornaviridae family. They are non-enveloped, and their genome is made up of single-stranded RNA. They are the agents of the common cold and can occasionally cause conjunctivitis through binding to the intracellular adhesion molecule 1 (ICAM-1), which is expressed at low levels throughout the body, including the ocular epithelia [52]. 

Coronaviruses are responsible for infections in both humans and other animals. All coronaviruses have a lipid membrane (envelope) surrounding their capsid (protein) which, in turn, contains RNA (genetic material) [59]. The proteins that protrude from the lipid envelope can be viewed under an electron microscope to simulate the shape of a crown that envelops the virus; the name of the entire family is derived from this morphology [60]. There are four main structural proteins typical of coronaviruses: the spike, the membrane, the envelope, and the nucleocapsid proteins. The spike protein allows the virus to attack the host cell receptors, with consequent fusion and viral entry. The membrane protein represents the most abundant viral component and defines the shape of the viral envelope. The envelope protein participates in the assembly of new virions and in budding from the host cell. Finally, the nucleocapsid protein binds to the viral genome and is also involved in viral assembly and budding [59]. Coronaviruses in the last 20 years have been the protagonists of several fatal infections in humans; specifically, we should mention severe acute respiratory syndrome coronavirus 1 (SARS-CoV) in 2002–2003, Middle East respiratory syndrome (MERS-CoV) in 2012 and, of course, severe acute respiratory syndrome coronavirus 2 (SARS-CoV-2) in 2019 [12,61]. 

SARS-CoV-2 is caused by a novel human-to-human airborne *Betacoronavirus*, with a genome consisting of single-stranded positive-sense RNA (+ssRNA) larger than that of any other RNA virus [62]. In December 2019, SARS-CoV-2 emerged in the Hubei region of China and caused an outbreak in the city of Wuhan. After a few months, the disease—named coronavirus disease 2019 (COVID-19)—had also spread quickly in Italy and Europe. On 11 March 2020, the World Health Organization (WHO) declared COVID-19 a pandemic. Since then, the cumulative number of confirmed cases worldwide has reached 65,870,030 with 1,523,583 deaths as of 6 December 2020 [63].

The transmission of SARS-CoV-2 occurs via direct contact from person to person, through airborne droplets or, more rarely, following contact with contaminated surfaces. SARS-CoV-2 is capable of binding the angiotensin-converting enzyme 2 (ACE2), facilitating infection in humans [64]. Conjunctival involvement (in the form of conjunctival hyperemia) is present in about 1% of patients [65]. The connection between SARS-CoV-2 infection and conjunctivitis is not well known to scientists at present. However, there are works that highlight the possibility of early ocular manifestations, so it is necessary to consider COVID-19 infection that initially manifests itself as viral conjunctivitis [66]. To date, it is very likely that these cases of conjunctivitis caused by SARS-CoV-2 have gone unnoticed because they were asymptomatic, so they did not involve cough, fever, and/or respiratory symptoms. Occasionally, there have been cases of conjunctivitis in confirmed COVID-19 patients [67]. Cases have been reported, such as the case of a patient with viral keratoconjunctivitis reported by Cheema et al., where the SARS-CoV-2 infection only affected the ocular surface. According to some scholars, such as Sun et al., the eye is rarely involved in human CoV infections; however, all cases of (kerato)conjunctivitis should be treated as potential COVID-19 cases—particularly those with severe symptoms [68]. Conjunctival epithelial cells can serve as the gateway and replication site for the SARS-CoV-2 virus prior to secondary upper airway contamination via the tear ducts [69]. Additionally, COVID-19-associated conjunctivitis is accompanied by the shedding of infectious viral particles in the tears [24,70]. These data should recommend the use of eye protection (e.g., goggles, visors) by exposed persons. To date, conjunctivitis and keratoconjunctivitis constitute the majority of SARS-CoV-2-related ocular infections, as widely reported in the literature. A recent study reported the existence of benign and asymptomatic retinal damage. As for other potential forms of eye damage, the lack of perspective still does not allow for any certainty [71,72].

#### 3.1.2. Herpesviruses

Herpesviruses are classified into three subfamilies: the alpha-herpesvirinae, the beta-herpesvirinae, and the gamma-herpesvirinae. Herpes simplex virus 1 and 2 and varicella-zoster virus belong to the first group; cytomegalovirus and human herpesvirus 6 and 7 belong to the second group; and the Epstein–Barr virus and human herpesvirus 8 belong to the third group.

The structure of herpesviruses is characterized by a rounded virion consisting of a capsid, integument, and envelope. Their genome is a linear double-stranded DNA molecule.

Herpesviruses can cause a wide range of diseases, and many of them can involve the eyes. Ocular infections caused by herpesviruses are very frequent. Post-herpetic neuralgia in the trigeminal area can be particularly debilitating. Other herpesviruses (e.g., HSV, CMV) can occasionally cause necrotizing retinitis, which must be considered to be an absolute visual emergency [73].

Herpes simplex virus type 1 (HSV-1) is responsible for ocular, labial, and oropharyngeal manifestations. Herpes simplex virus type 2 [74,75] (HSV-2), on the other hand, is mainly responsible for genital manifestations. However, both have the ability to induce manifestations in the same regions of the body. 

HSV-1’s ocular manifestations mainly cause infectious blindness. HSV-1 infection can affect any part of the eye but more often causes epithelial or dendritic keratitis. Herpes stromal keratitis can cause corneal opacification and loss of vision [72]. The consequences of the infection are due both to the viral pathogenesis and to the immunopathogenesis that occurs following the response of the host cell to the virus. The capsid, in turn, is covered by an external lipid bilayer containing glycoproteins (i.e., gB, gC, gD, gH, and gL) that interact with receptors located on the surface of the host cell [76]. This interaction facilitates the penetration of the capsid through the host’s plasma membrane. The heparan sulfate, through its interaction with gB and/or gC, enables the attachment of the virus to the cells, followed by the binding of the viral gD with 3-OS heparan sulfate or nectin-1, necessary for the release of the capsid in the cytoplasm [77]. The virus enters the cell in different ways depending on the type of cell, e.g., merging with the membrane of the host cell, or through endocytosis [78]. The capsid is then transported to the nucleus for viral genome replication using the host cell’s DNA polymerase. The new virions produced are released from the cell to infect neighboring cells. HSV can replicate by infecting nearby cells or enter a latent state, reaching the trigeminal ganglion via the cornea. Reactivation from the state of latency is responsible for the persistence of the infection throughout the life of the host [79]. Primary HSV infection often occurs in children or young adults through direct contact with infected mucous membranes and causes conjunctivitis with eyelid blisters. Most cases of ocular HSV are due to reactivation of latent HSV infection from the trigeminal ganglion through viral spread to the cornea. Reactivation of HSV has been observed to depend on various environmental factors, including stress, fever, and exposure to ultraviolet light, as well as iatrogenic factors such as laser treatment or topical use of corticosteroids [80]. In 60% of cases, ocular HSV infection presents with corneal epithelial disease, progressing from punctate keratitis to dendritic keratitis or geographic ulcer [81]. Patients initially present with symptoms of eye pain, tearing, redness, and foreign body sensation. Dendritic keratitis is characterized by branching lesions on the corneal epithelium, leading to loss of or reduction in corneal sensation [82]. Although epithelial lesions can resolve on their own, antiviral therapy is used to accelerate the resolution of these lesions and limit the extent of the disease [83]. Herpes stromal keratitis (HSK) is associated with recurrent infections and leads to long-term loss of vision due to the sequelae of corneal scarring and neovascularization. In fact, at each recurrence of HSK, there is a further stromal immune response in the cornea, with progressive opacification and neovascularization [84]. Several forms of HSK have been reported, including necrotizing stromal keratitis and non-necrotizing or disciform immune stromal keratitis. The former is characterized by the presence of a stromal infiltration with or without epithelial ulceration, perhaps following a viral invasion of the corneal stroma; therefore, corneal fusion can occur due to the important inflammatory response within the cornea [85]. 

The latter, on the other hand, is the most common manifestation of HSK and presents with stromal inflammation without necrosis. HSV endotheliitis can also be described as disciform keratitis with focal stromal edema and keratin precipitates. HSV was identified by PCR from aqueous samples from patients with endotheliitis [86]. Infection can also cause anterior chamber inflammation with endotheliitis or in isolation as HSV uveitis [87]. Upon examination, patients may present with elevated intraocular pressure due to inflammation of the trabecular network that responds to topical corticosteroids [81,88]. 

The host cell responds to HSV infection with a complex series of immunopathogenetic events, manifesting as epithelial, stromal, or endothelial keratitis [81,88]. Innate and adaptive immune responses are responsible for eliminating active HSV infection, but they also produce damaging inflammation within the cornea that can lead to neovascularization and scarring [89]. Herpesviruses reach the ocular surface from the external environment and are initially suspended in the tear film [90]. Several factors intervene in preventing viral infection of the surface of the eye. First, the tear film contains various components, such as IgA, complement factors, peroxidase, interferons, etc., which act by preventing the action of viral particles. In addition, the ocular surface is subjected to continuous washing by the tears produced by the lacrimal gland, allowing the efficient removal of viral agents [91]. This first line of defense is not enough to protect the eye from viral attack. The outer cells of the cornea are cells in the terminal stage of replication and are constantly replaced by those below, removing the cause of the infection and limiting its replication [92]. These mechanisms, taken together, work to prevent viral infections. During a viral infection, an inflammatory reaction occurs, induced by polymorphonuclear leukocytes, macrophages, and a dispersion of other mononuclear cells, such as lymphocytes and natural killer cells [93]. TRL receptors 2, 3, and 9 detect viral products (i.e., specific molecular models associated with pathogens—“PAMPs”). In particular, TLR2 binds glycoproteins to the cell surface, while TLR3 and TLR9 in endosomes recognize double-stranded RNA and non-methylated CpG motifs, respectively [94]. The binding of TRLs and PAMPs not only intervenes in viral clearance but also triggers a cascade of events that lead to the production of cytokines and chemokines that attract T lymphocytes to the lesion, where they recognize the antigens presented by major histocompatibility complexes (MHCs) [95]. Among the first cytokines secreted following HSV-1 infection of the cornea are type I interferons (i.e., IFN-α and IFN-β), which are produced by several cell types, including corneal epithelial cells. IFN-α and -β are necessary for the correct control of HSV-1 replication in the cornea and for the correct recruitment of immune cells (e.g., leukocytes) to the site of infection [96]. Interferon-γ also plays an important role in controlling infection. It is produced only by cells of the immune system—especially Th1 cells—and increases cytokine production, phagocytosis, and the expression of class II MHCs [97]. Additionally, the pro-inflammatory cytokines IL-1, IL-6, IL-17, and TNF-a contribute to corneal inflammation [98,99]. IL-6 and IL-17 intervene by increasing the production of VEGF, which is involved in corneal vascularization. Furthermore, CXCL1 and CXCL2 guide neutrophils to the site of infection [100]. Although many cytokines promote leukocyte infiltration and tissue damage, others play a protective role. IL-10 has been shown to attenuate immune responses. It is produced by resident corneal cells and reduces the levels of IL-2 and IL-6, as well as the infiltration of neutrophils in the cornea, with consequent reduction in its opacity [101]. Several studies have also shown that the enzyme heparanase (HPSE), when produced by the host cell, promotes HSV infection and pathogenesis through the following mechanisms: (1) HPSE contributes to viral spread by removing heparan sulfate from the cell surface and allowing newly manufactured virions to be released into the surrounding environment; (2) HPSE participates in tissue damage and corneal inflammation by releasing growth factors such as VEGF from the cell matrix; (3) finally, HPSE appears to increase the transcription of pro-inflammatory cytokines such as IL-1β, IL-6, and TNF-α [92]. Therefore, HPSE may represent a therapeutic target for controlling the pathogenesis of HSV infection and corneal inflammation [102]. Immunopathological damage, together with the ability of the virus to evade certain mechanisms of the host immune response—including downregulation of TLR, of the RLR receptor (RIG-I-like), and of cytosolic DNA detection; the expression of viral kinases capable of altering the phosphorylation status of transcription factors to interfere with the nuclear translocation and expression of target genes, as in the case of the regulatory factor of interferon 3 (IRF3) and nuclear factor (NF)–κB; and the destruction by the virus of important cellular defense mechanisms such as MHC class I antigen presentation, DNA damage response pathways, autophagy, endoplasmic reticulum stress, and necroptosis—is capable of promoting the propagation and persistence of the virus [102]. As for the acquired immune response, it begins when the cells presenting the viral antigen (i.e., Langerhans cells and macrophages) migrate to the regional lymph nodes, where they activate the T lymphocytes. The HSV infection that occurs in the cornea determines the establishment of systematic immunity in the infected individual.

Therefore, improving the knowledge of these host factors and strengthening these cellular responses can yield valuable therapeutic options.

The varicella-zoster virus (VZV) is responsible for two clinically distinct forms of disease. The primary infection manifests itself as chickenpox, characterized by skin rashes all over the body and by a permanent infection of the neurons of the sensory ganglia. Reactivation, on the other hand, manifests itself as herpes zoster—a neurocutaneous disease characterized by painful one-sided vesicular rashes. The eye can be involved both during primary infection and reactivation [103,104].

The viral envelope originates from host cells and then dissolves with the use of detergents. Using molecular techniques, it was possible to identify five different genotypes of VZV, including B, C, J, J2, and A; the first two are found predominantly in the United States and Europe, while the others are found in Africa and Asia [105]. The virus is transmitted by direct contact between cells, and its site of entry is the upper respiratory tract, where the replication of viral particles begins [106]. Subsequently, following lymphatic diffusion of the viral particles from the nasopharynx to the circulating T lymphocytes, the virus circulates in the blood [107]. It eventually reaches the skin by infecting the epithelial cells and causing chickenpox [36]. The varicella-zoster virus reactivates “spontaneously” or following triggers. Most cases of HZO present with fever, malaise, headache, and eye pain. Recent studies report that ocular involvement occurs in approximately 50% of patients with HZV infection [108]. Herpes zoster ophthalmicus (HZO) causes serious damage to the cornea after an acute infection in approximately 65% of patients, and it is difficult to precisely establish the various factors potentially involved in corneal damage [82]. The corneal involvement includes punctate epithelial keratitis, early pseudodendrites, anterior stromal infiltrates, corneal mucosal plaques, disciform keratitis, neurotrophic keratitis, and/or exposure keratitis [81]. HZO also causes conjunctivitis, which can induce a pseudomembranous, membranous, or follicular response, often associated with petechial hemorrhages and blisters on the bulbar or eyelid conjunctiva [109]. Uveitis may occur at the end of chickenpox, but more frequently occurs with ophthalmic zoster [110]. Anterior uveitis is frequently unilateral, acute hypertensive, plastic, or granulomatous, but rarely bilateral. In the case of anterior uveitis, the inflammation presents with mild symptoms and resolves in the short term but can cause an increase in intraocular pressure (hypertensive uveitis) [111]. Zoster uveitis can cause iris atrophy and irregular pupil, and as a complication it can cause glaucoma, with an incidence of approximately 13.1% [112].

Cytomegalovirus (CMV) is a ubiquitous virus [113]. The main modes of transmission of the infection include contact with bodily fluids or breast milk, through sexual contact, and through organ transplantation. Primary CMV infection can occur at any age; however, in children, primary CMV infection may cause few symptoms or be asymptomatic, while in adults primary CMV infection generally causes fever associated with transient lymphocytosis and changes in liver function parameters [114]. During primary infection, the virus can reach all parts of the body, including the bone marrow. Following infection, the viral genome migrates and replicates in the nucleus of the infected cell. In cells that are permissive for viral replication, such as fibroblasts or endothelial cells, the transcription of early genes coding for and capable of regulating DNA-binding viral proteins occurs first, followed by the transcription of late genes that encode viral structural proteins, which are then assembled to form new viral particles [115]. Conversely, the virus becomes latent in the bone marrow, as infected myeloid progenitor cells inhibit the production of new viral particles. Reactivation of the virus is normally controlled by the immune system, but CMV can cause serious illness in immunocompromised patients [116]. The most serious ocular CMV infections are represented by retinitis and uveitis [117]. CMV retinitis occurs in patients who have failed to generate a primary T-cell response against the virus or in patients who are carriers of CMV, but whose CMV-specific T-cell response has been suppressed by disease or immunosuppressive treatments. Retinitis is often bilateral and requires prolonged antiviral treatment until the state of immunosuppression is reduced. The risk of CMV retinitis is greater in CMV-positive patients transplanted with bone marrow from a CMV-negative donor, and infection generally occurs within 12 months of transplantation [118]. In HIV infection, CMV retinitis generally occurs in advanced stages of the disease when CMV-specific T cells are functionally reduced [119]. In recent years, CMV infection has been associated with two other forms of anterior segment disease: uveitis and endotheliitis. Anterior uveitis can be acute or chronic and is usually unilateral with increased intraocular pressure; CMV corneal endotheliitis causes loss of corneal endothelial cells, local corneal stromal edema, and keratin precipitates, sometimes accompanied by increased intraocular pressure or iris atrophy [120]. Some studies have linked the development of CMV endotheliitis to previous local or systemic immunosuppression or penetrating corneal surgery. Since corneal endotheliitis can also be secondary to reactivation of HSV or VZV, accurate virological diagnosis by PCR amplification is required to ensure proper antiviral treatment [121]. 

#### 3.1.3. Arboviruses and Emerging Viruses

Arthropod-borne viruses (arboviruses) are a large and diverse group of viruses that are transmitted from an infected host to a healthy host through the bite of an arthropod vector, causing numerous deaths worldwide each year [122]. About 150 arboviruses are responsible for human diseases [123]. Among them, most belong to four families: Flaviviridae (genus *Flavivirus*), Togaviridae (genus *Alphavirus*), Peribunyaviridae (genus Orthobunyavirus), and Phenuiviridae (genus *Phlebovirus*) [124]. Arboviruses cause infections that often present with flu-like symptoms [125]. Consequently, it is often not easy to discriminate this causative agent from others, and the percentage of cases of infection can be underestimated. Arboviruses can cause ocular manifestations ranging from dengue fever virus (DFV) to hyposphagma and maculopathy [123]. They can cause conjunctivitis, uveitis, increased intraocular pressure (IOP), retinal vasculitis and, less frequently, hemorrhages [126].

Dengue: Dengue is a mosquito-borne viral disease that has rapidly spread to subtropical and tropical regions in recent years [127]. In particular, *A. aegypti* is the main vector responsible for the transmission of this disease. There are several serotypes of DFV—DENV1, DENV2, DENV3, and DENV4—and it became possible to verify the differences between them in 1940 and 1956 [128]. DENV infection presents with a wide range of clinical manifestations, from a mild flu-like syndrome known as dengue fever (DF), to life-threatening events such as dengue shock syndrome (DSS) [129]. The characteristic symptoms of FD are fever, nausea, vomiting, and rash, while in DSS severe bleeding and shock can occur and, if not adequately treated, mortality reaches 20% [130]. 

This arbovirus causes conjunctival petechiae and inflammatory disease of the posterior segment of the eye (maculopathy) in 50% of cases, sometimes in conjunction with anterior uveitis [131]. Typically, bilateral vision loss (usually of the scotoma type) occurs within 8 days of the onset of fever and spontaneously regresses within a few weeks [132]. The most common signs are hyposphagma (37% of cases), uveitis (albeit rarer), and increased intraocular pressure. Dengue-related ophthalmic complications are not particularly problematic in most cases and heal without treatment [133]. In more severe cases, such as those involving loss of vision, systemic steroids are used. Retinal manifestations in DFV occur in the vascular system of the posterior segment of the eye. Maculopathy, characterized by vasculitis and hemorrhages, is the main retinal manifestation and is generally bilateral (73% of cases) [123]. Other known manifestations of the posterior segment include retinal venous enlargement; increased upper retinal vascularity and vascular sheath; the presence of tortuous vessels; acute macular neuroretinopathy; intraretinal, peripheral, or peripapillary macular hemorrhages; retinal edema (macular and diffuse); Roth staining; choroidal effusions; choroidal neovascularization; swelling of the optic disc; and neuropathy of the optic disc [134].

Chikungunya: The manifestations affecting the anterior and posterior segment of the eye are infrequent in patients with Chikungunya infections. The limited evidence reported describes symptoms such as photophobia, retrobulbar orbital pain, and conjunctivitis [135]. The main ocular manifestation is anterior uveitis [126]. 

Zika virus (ZIKV): The Zika virus (ZIKV)—a mosquito-transmitted Flavivirus—takes its name from the Zika Forest located in Uganda, where it was first isolated in rhesus monkeys in 1947 [136]. The virus then migrated to Asia during the 1940s and has caused outbreaks outside Asia in the last two decades, in countries such as Micronesia, French Polynesia, and Chile’s Easter Island. However, the most recent ZIKV outbreak dates to 2015 in Brazil [123]. Wang et al. showed that ZIKV typically leads to mild symptoms in infected adults, but that the infection can cause fetal brain injury in pregnant women infected by the virus [137]. The infection is transmitted to humans through the bites of *Aedes* spp. After transmission, the initial phase of infection is characterized by rapid and acute amplification of the virus in peripheral tissues, followed by its elimination by the host’s immune response. The acute phase is associated with non-purulent conjunctivitis and with inflammatory ocular manifestations such as anterior uveitis in infected adults [126]. However, ZIKV poses an emerging threat to pregnant women across much of the Americas, Asia, and Africa. ZIKV infection during pregnancy has been associated with congenital microcephaly. Infection in the fetus is the result of a complex series of events that begin with viral infection, the elusion of the host’s immunity, and the ability of the virus to overcome the barriers of placental and fetal tissue [138]. Evasion of host immunity is a key event that facilitates ZIKV infection, viral propagation, and spread to the fetus. ZIKV is able to deploy several mechanisms to evade antiviral immunity [139]. These defensive strategies involve viral proteins, genomic RNA elements, secreted particles, and processes that occur during viral replication [140]. However, to date, the mechanisms by which ZIKV infection and host immune evasion occur in the context of the relevant tissues and cell types encountered during viral trafficking in the brain and cells are not fully known [141]. Once ZIKV has entered the fetal circulation, it is likely to cross both the blood–CSF (choroid plexus) and the blood–retina barriers [139]. The likelihood of congenital ZIKV infection and resultant microcephaly is greatest during the first trimester of pregnancy, when fetal immunity is likely to be highly compromised. It is recommended to perform a fundus in all infants with possible maternal–fetal ZIKV infection [142]. 

West Nile fever: The most frequent manifestations of this arbovirus are multifocal chorioretinitis and neuroretinitis (often associated with retinal vasculitis), which are very common in an epidemic context [143]. 

Ebola viruses: Belonging to the genus *Ebolavirus*, these are non-segmented, negative-stranded RNA viruses that cause a severe hemorrhagic fever [144]. Five species of Ebola viruses are known: Bundibugyo, Zaire, Sudan, Reston, and Tai Forest [145]. The first three are responsible for epidemics in Africa with the highest rates of mortality. Ebola virus disease (EVD) is a zoonosis. The natural reservoirs of the virus are likely fruit bats, as the virus has often been found in these animals with no signs or symptoms of infection [146]. The disease can be transmitted to humans through contact with the blood or bodily fluids of infected animals. Ebola viruses infect human monocytes that release chemokines and pro-inflammatory cytokines, leading to the loss of the endothelial barrier function [147]. This mechanism, together with the ability of the virus to evade the host’s innate, humoral, and cellular immune responses, can promote rapid viral dissemination. The most common manifestations of EVD are fever, asthenia, myalgia, diarrhea, abdominal pain, rush, melena, and conjunctival infections [148]. Clinical conditions may worsen to death from anuria, shock, hiccups, dysesthesia, and bleeding. About 20% of convalescent patients develop uveitis with photophobia, hyperlacrimation, and loss of vision [149]. The major cause of uveitis is a hypersensitivity to viral antigens. In the acute phase, it is often responsible for conjunctival hyperemia, the pathophysiology of which is unknown. Visual impairment is most associated with posterior uveitis; however, untreated disease can also be associated with structural complications, including cataracts and vitreous opacity [148]. Cataracts and vitreous opacities represent identifiable causes of vision loss and have medical and surgical implications, particularly considering the discovery of live Ebola virus in the ocular fluids of Ebola survivors [24]. Given the possible persistence of EBOV in ocular fluid, further studies are needed to evaluate the persistence time of the Ebola virus in ocular fluids. In addition, a better understanding of the pathogenesis associated with the different types of ocular inflammatory diseases manifested following infection should also include an assessment of the biomarker levels of ocular and systemic inflammation [150]. This will allow the formulation of an appropriate medical therapy and the adoption of valid precautions to allow the protection of ophthalmologists, patients, and the families of both. The convalescence phase can be characterized by potentially serious inflammatory manifestations in almost 15% of patients (e.g., anterior and posterior uveitis, panuveitis, optic neuritis), with a risk of losing sight of around 40% [151].

#### 3.1.4. Other Viruses

In a low percentage of cases, *Molluscum contagiosum* (MC) infection, characterized by multiple umbilicate and papular skin lesions caused by the Pox-2 virus, causes primary lesions in the conjunctiva [152]. MC is contracted following skin-to-skin contact and sexual intercourse. The dispersion of viral proteins from the eyelid lesions into the tear film leads to a chronic follicular conjunctival reaction [153].

## 4. Antiviral Treatments in Ocular Pathologies: What Is Ongoing and What Is New?

Antiviral effects are exerted by chelating, buffering, or oxidative agents commonly used in the formulations of eye drops, such as sodium perborate, sodium bicarbonate, citric acid, boric acid, and stabilized oxychloro complex [154,155]. Among these, BAK (a quaternary ammonium salt derivative) has demonstrated virucidal activity against all lipid-enveloped viruses, although it does not always seem to be efficient against SARS-CoV-2 [156]. Among the disinfectant agents included in ophthalmic formulations, sodium hypochlorite [157,158] is able to inactivate mouse hepatitis virus [159]. European regulations also recommend sodium hypochlorite (at least 0.21%) with povidone iodine (>0.75% free iodine) for use against ocular infections caused by SARS-CoV-2 [160]. Antiviral action against many RNA or DNA viruses is exerted by different substances contained in artificial tears [161]. Particularly, some viruses belonging to the Coronaviridae family are inhibited by natural extracts (e.g., *Ginkgo biloba*) and polymer constituents, such as high-molecular-weight hyaluronic acid [162]. Interestingly, zinc (0.25%)—an electrolyte used as an astringent or excipient in artificial tears—is able to block SARS-CoV-2’s viral replication by inhibiting its polymerase activity [163]. Moreover, a variable degree of antiviral action is showed by common excipients with antioxidant activity included in artificial tears, such as acetylcysteine and vitamins A, C, and D [160]. Particularly, vitamin D enhances the therapeutic antiviral response to recurrent hepatitis C virus [164]. Finally, a mild virucidal action is hypothesized for other ingredients present in artificial tears, such as L-carnitine, glycerol, and ozonated oils [165]. A recent randomized clinical trial showed that the use of eye drops containing ozonated oil along with the combination tobramycin 0.3%/dexamethasone 0.1% reduces the signs of conjunctivitis and the duration of viral infection, without affecting the appearance of subepithelial corneal infiltrates [166]. Among drugs that have a repurposing potential for their antiviral activity, chloroquine has shown good efficacy against flaviviruses, retroviruses and coronaviruses, including SARS-CoV-2. Indeed, this antimalarial drug is able to inhibit the pH-linked steps of viral replication, and it has recently been included in eye drop formulations at a concentration of 0.03% [167]. Moreover, glaucoma drugs may have a synergistic action against viral infections—for example, timolol maleate is used for the treatment of herpes simplex infections [168]; brinzolamide is able to achieve a moderate inhibition of the H3N2 and H1N1 influenza viruses, along with a weak inhibition of the H5N2 and H7N1 influenza viruses; and dorzolamide has emerged as an inhibitor of oseltamivir-resistant influenza viruses [166,167]. Moreover, antiviral activity is shown by a limited number of antibiotics, such as fluoroquinolones, which are effective against influenza viruses and polyomavirus BK [169]; chloramphenicol, which is effective against the human Herpesviridae family [170]; fusidic acid, which is effective against human immunodeficiency virus and John Cunningham virus infection [171]; aminoglycosides, which are effective against Japanese encephalitis and influenza A virus infection [172,173]; and colistin, which is effective against mycobacteriophage D29 infection [174]. For the specific treatment of COVID-19, tetracyclines have been proposed for their antiviral potential, while macrolides have shown efficient antiviral activity [175,176]. Although rarely used in ocular pathologies, antiviral effects have also been shown by some antifungals (e.g., posaconazole, amphotericin B, and itraconazole) [160]. Interestingly, several anti-allergic eye drops—frequently used as treatments in ophthalmology—have shown antiviral activity against Ebola viruses, cuevavirus, Marburg virus, and Influenza A virus, particularly due to antihistamines and diphenhydramine. Similarly, antiviral effects against *polio-virus* type 1, HSV-1, and respiratory syncytial virus (RSV) have been observed with the use of flavonoids in allergic eye disorders, while topical immunomodulators such as cyclosporines reduce the growth of Flavivirus, HCV and influenza virus [177]. Finally, by counteracting the inflammatory response and inhibiting herpetic viruses, both non-steroidal anti-inflammatory drugs (NSAIDs) and steroids show antiviral effects [160]. Particularly, NSAIDs lower the levels of prostaglandins associated with latent HSV activation, while dexamethasone could be used for viral conjunctivitis [178]. 

Important advances have been made in antiviral chemotherapy. Nine systemic drugs (vidarabine, aciclovir, famciclovir, valaciclovir, ganciclovir, valganciclovir, cidofovir, foscarnet, and fomivirsen) and four topical agents (idoxuridine, vidarabine, trifluridine, and aciclovir) are exploited in ocular infections of viral etiology. The viral agents targeted by these drugs are HSV, VZV, CMV, vaccinia virus, adenovirus, and EBV (Table 2) [50,179]. An ideal antiviral agent interrupts the replication cycle of the virus without affecting the host cell. Adsorption, penetration, replication of genetic material, and transcription of the viral genome are the main processes that must occur for the infectious process to be successful. Current therapy focuses on these functions in order to stop the generation of new virions. Among the drugs widely used in the treatment of eye infections are idoxuridine, vidarabine, trifluridine, and acyclovir [180].

Idoxuridine is a thymidine analog with a methyl group substituted by iodine in the 5′ position. This drug is phosphorylated by both viral and cellular kinases. Idoxuridine monophosphate can be incorporated as a thymine analog in viral DNA, resulting in impaired transcription and translation, thereby producing defective viral progeny. Idoxuridine triphosphate inhibits DNA polymerase and dTMP synthetase, disrupting their function [181]. This drug is used topically only for HSV keratitis. It is not effective in the treatment of HSV-induced iritis, stromal keratitis, or other forms of intraocular infection [182]. Idoxuridine is teratogenic, mutagenic, and potentially carcinogenic; for these reasons, it is not used systematically. Adverse reactions that develop after topical use include contact dermatitis, punctate epithelial keratopathy, follicular conjunctivitis, tear point stenosis and occlusion, and lid margin keratinization. It is among the most toxic drugs; hence, with the development of new, less-toxic drugs, it is no longer used [183]. Viral resistance to idoxuridine occurs through modification of the gene encoding thymidine kinase [184]. Resistance to idoxuridine develops easily under laboratory conditions. Viral strains resistant to this drug are frequently isolated from patients with keratitis treated with idoxuridine [183].

Trifluridine is a thymidine analog with a methyl group substituted by three fluorine atoms in the 5′ position [185]. The drug is initially phosphorylated to trifluridine monophosphate and acts as an inhibitor of thymidylate synthase. In its triphosphorylated form, it competitively inhibits the incorporation of thymidine triphosphate into viral DNA [186]. Trifluridine blocks the replication of herpesviruses and vaccinia viruses [187]. The use of this drug is limited to topical application due to its cytotoxicity, teratogenicity, mutagenicity, and carcinogenicity. In vivo studies in mice did not find adverse effects or evidence of corneal toxicity [188]. Resistance to trifluridine is rare and occurs through modifications of the enzyme thymidylate synthetase [189]. Strains resistant to trifluorothymidine have been selected in vitro. However, significant resistance rates have not been established [186].

Vidarabine is a purine arabanosyl nucleoside [190]. This drug also undergoes single, double, and triple phosphorylation in the host cell’s cytoplasm. Vidarabine inhibits the formation of new viral particles through several mechanisms. It causes DNA chain termination through incorporation into viral DNA and inhibits terminal deoxynucleotidyl transferase, DNA polymerase, and ribonucleotide reductase enzymes [190,191]. Vidarabine acts on a broad spectrum of viruses, including HSV-1, HSV-2 VZV, CMV, vaccinia virus, and pseudorabies virus. This drug is highly effective against HSV-1 and HSV-2 [192]. In a study of patients with external ocular herpetic keratitis, vidarabine significantly reduced viral titers, promoting corneal re-epithelialization. Its systemic use has been shown to be effective in eye infections caused by VZV. Treatment with vidarabine, at a dosage of 10 mg/kg/day for 5 days, reduced the viral titers, the febrile period, and the ocular lesions compared to the control group [193]. The drug shows teratogenicity, mutagenicity, and carcinogenicity; therefore, it is used systematically only as an alternative to acyclovir or ganciclovir in the event of resistance. Its topical administration to rabbits did not alter their ocular function [194]. Vidarabine-resistant viruses with gene changes in the polymerase DNA have been isolated that make viral DNA polymerase less susceptible to vidarabine. The degree of resistance to vidarabine is four times lower than the resistance recorded for acyclovir [195]. 

Acyclovir is an analog of guanosine characterized by the lack of carbon atoms in the 2′and 3′ positions and is one of the most widely used antivirals against herpesviruses, thanks to its high selectivity and few side effects [196]. In fact, all herpesviruses share some significant biological properties in the replication cycle—they specify a wide range of enzymes involved in the metabolism of nucleic acids, in the synthesis of DNA, and in the post-translational processing of proteins. Aciclovir acts through one of these enzymes—thymidine kinase, which is encoded only by herpetic viruses and, therefore, is absent in healthy cells and is very selective. In particular, the drug is phosphorylated into acyclovir monophosphate by herpesvirus-encoded thymidine kinase, and then it is further phosphorylated to acyclovir diphosphate and triphosphate by viral and cellular kinases. Acyclovir triphosphate is irreversibly incorporated into the viral DNA-virus-specific DNA polymerase, causing the replication of the viral genome to be blocked. Acyclovir exhibits effective action on HSV-1 and HSV-2, VZV, EBV and, to a lesser extent, CMV [197]. This drug is approximately 160 and 10 times more effective than vidarabine and idoxuridine, respectively [193]. Acyclovir has been shown to suppress latent virus reactivation in explanted trigeminal ganglia but does not eliminate latency. This is evidenced by the increase in viral titers after discontinuation of treatment [198]. Acyclovir shows no significant degree of toxicity. No ocular toxic effects were noted after topical application [199]. Resistance to acyclovir is uncommon but occurs more frequently in immunocompromised patients. Resistance to acyclovir occurs through mutations in the thymidine kinase gene, also conferring cross-resistance to antiviral nucleoside analogs such as valaciclovir, famciclovir, and penciclovir [200]. Prevalence rates were 0.5% in immunocompetent patients and 5–6% in immunocompromised individuals [201].

## 5. Conclusions

The ocular structure is susceptible to viral infections, with implications ranging from mild to severe discomfort. Antiviral drugs can be used for prophylaxis and treatment of infections. Two important factors limit the usefulness of antiviral drugs: toxicity, and the development of resistance to the antiviral agent. Corticosteroids are useful for modulating the immune reaction of the eye, limiting the damage. The emergence of ocular infections that are resistant to antiviral drugs stimulates the scientific community to search for novel therapeutic strategies to address these important infections.

## Figures and Tables

**Table 1:** Sites of ocular infections and associated etiological agents.

Site	Infection	Etiological Agents
**Ocular surface**	Conjunctivitis	Adenovirus, HSV, *Staphylococcus* spp., *Streptococcus* spp., *Clamydia trachomatis, Neisseria gonorrhoeae*
	Keratitis	HSV, VZV, *Pseudomonas aeruginosa*, *Staphylococcus* spp., *Acanthamoeba* spp., *Candida* spp., *Aspergillus* spp.
	Episcleritis/scleritis	VZV, *Mycobacterium tubercolosis*
**Internal eye**	Anterior uveitis	HSV, VZV, CMV
	Intermediate and posterior uveitis	HSV, VZV, CMV, *Mycobacterium tubercolosis, Toxoplasma gondii, Treponema pallidum*
	Endophthalmitis	*Staphylococcus* spp., *Streptococcus* spp., *Escherichia coli*, *Pseudomonas aeruginosa, Candida* spp., *Aspergillus* sp., *Fusarium* spp.
**Eye annexes**	Blepharitis	*Staphylococcus* spp., *Demodex folliculorum*
	Dacryocystitis	*Staphylococcus* spp.

**Table 2:** FDA-approved antiviral agents for viral ophthalmic infections.

Antiviral	Chemical Structure	Mechanism of Action	Target Viruses
Trifluridine	Pyrimidine nucleoside	Inhibits thymidylate synthetase	HSV-1, HSV-2, vaccinia virus
Vidarabine	Purine nucleoside	Inhibits multiple enzymes (e.g., DNA polymerase)	HSV-1, HSV-2 VZV, CMV, vaccinia virus, pseudorabies virus
Acyclovir	Acyclic pyrimidine nucleoside	Viral DNA chain termination; inhibits viral DNA polymerase	HSV-1, HSV-2, VZV, EBV, CMV
Valaciclovir	1-Valine ester of acyclovir	Viral DNA chain termination; inhibits viral DNA polymerase	HSV- 1, HSV-2, VZV, EBV, HHV-6, HHV-8, CMV
Famciclovir	Acyclic guanine derivative	Viral DNA chain termination; inhibits viral DNA polymerase	HSV- 1, HSV-2, VZV, EBV
Ganciclovir	Acyclic pyrimidine nucleoside	Viral DNA chain termination; inhibits viral DNA polymerase	CMV, HSV-1, HSV-2, VZV, EBV
Valganciclovirn	Monovalent ester of ganciclovir	Viral DNA chain termination; inhibits viral DNA polymerase	CMV, HSV-1, HSV-2, VZV, EBV
Idoxuridine	Pyrimidine nucleoside	Inhibits viral DNA thymidine uptakes, viral DNA polymerases, and viral DNA incorporation	HSV-1, HSV-2
Cidofovir	Phosphonoformic acid derivative	Inhibits viral DNA polymerases	CMV, VZV, HSV-1, HSV-2 adenovirus, EBV, vaccinia virus
Foscarnet	Phosphonoacetic acid derivative	Inhibits viral DNA polymerases and RNA polymerases	CMV, HSV-1, HSV-2, VZV, EBV
Fomivirsen	Antisense oligonucleotide	Viral mRNA binding	CMV

## Data Availability

Not applicable.
